# Bibliometric analysis of the research on anti-Müllerian hormone and polycystic ovary syndrome: current status, hotspots, and trends

**DOI:** 10.3389/frph.2025.1519249

**Published:** 2025-04-24

**Authors:** Bingqing Ran, Cai Liu, Yajun He, Lizhu Ma, Fang Wang

**Affiliations:** ^1^ School of Integrated Chinese and Western Clinical Medicine, Gansu University of Chinese Medicine, Research Experimental Center, Gansu Province, Lanzhou City, China; ^2^ Lanzhou University, School of Clinical Medicine, Gansu Province, Lanzhou City, China; ^3^ The Second Hospital, Lanzhou University, Department of Reproductive Medicine, Gansu Province, Lanzhou City, China

**Keywords:** bibliometric analysis, CiteSpace, VOSviewer, AMH, PCOS

## Abstract

**Background:**

Polycystic Ovary Syndrome (PCOS) is a common endocrine and metabolic disorder affecting women of reproductive age. Over the past 30 years, significant efforts have been devoted to exploring its various pathogenic mechanisms, physiological and pathological characteristics, and biomarkers. Among these, Anti-Müllerian Hormone (AMH), as a biomarker for PCOS, is a significant biomarker for diagnosing, treating, and monitoring. However, the individual key information extracted from numerous studies is difficult to apply in clinical practice. Therefore, this article employs bibliometric analysis to summarize the current state of knowledge and offer future perspectives.

**Methods:**

The Science Citation Index Expanded (SCI-E) within the Web of Science Core Collection database has been identified as the material source for obtaining articles related to AMH and PCOS. Software such as Origin, Microsoft Excel, Pajek, VOSviewer, and CiteSpace were used for bibliometric analysis and statistical assessment, evaluating countries, institutions, journals, references, and authors, as well as for constructing visual knowledge network maps.

**Results:**

From 1994 to 2024, a total of 1,082 articles were included in the bibliometric analysis of research on AMH and PCOS. The number of publications in this field has consistently increased, with contributions from 70 countries, 1,363 institutions, and 5,144 researchers worldwide. Among them, the United States and China are the two countries with the highest number of publications. Zhejiang University, Monash University, and Peking University rank among the top three institutions exhibiting explosive citation bursts. The author with the highest publication volume is Didier Dewailly. The predictive keywords associated with these articles include “consensus,” “morphology,” “criteria,” “prevalence,” and “Müllerian hormone.”

**Conclusions:**

Through bibliometric analysis, this study has identified the primary research hotspots in the field of AMH and PCOS as follows: (1) Refining the diagnostic criteria for PCOS by using AMH as a biomarker; (2) Exploring the molecular role of AMH in the pathophysiological processes of various PCOS phenotypes and its potential as a therapeutic target; (3) Analyzing the impact of baseline AMH levels on female reproductive health and other biomarkers; (4) Investigating the signalling mechanisms of AMH in PCOS and its role in disease progression.

## 1 Introduction

Polycystic ovary syndrome (PCOS), first described by Stein and Leventhal in the 1930s, affects 11%–13% of reproductive-aged women worldwide, manifesting as oligo-anovulation, hyperandrogenism, and polycystic ovarian morphology (PCOM) (1). Its pathophysiology involves complex interactions between endocrine dysregulation (e.g., elevated LH/FSH ratio, insulin resistance) and epigenetic modifications in ovarian granulosa cells (such as AMHR2 promoter hypomethylation affecting gene expression and cellular function) (2, 3), and follicular microenvironment alterations (4). Anti-Müllerian hormone (AMH) has emerged as a pivotal biomarker, reflecting both ovarian reserve and follicular arrest severity in PCOS (5). The 2023 International PCOS Guideline endorses AMH as a surrogate for PCOM diagnosis via ultrasound, with ethnicity-specific thresholds recommended due to significant racial disparities. Notably, Japan's 2024 diagnostic criteria (JSOG2024) recalibrate AMH cutoffs for low-obesity phenotypes, underscoring the necessity of population-specific adaptations (6). A 2024 multicenter trial then confirms that AMH-guided ovarian stimulation significantly reduces OHSS risk by nearly half in Japanese IVF patients compared to standard dosing without compromising the live birth rate (7). This trial highlights the clinical utility of AMH in specific populations, prompting further exploration of population-specific AMH applications. Critical challenges persist in AMH clinical translation, particularly in low-resource settings where widespread clinical use of AMH testing coexists with inadequate standardization. While recent trials validate AMH as a cost-effective biomarker demonstrating a strong correlation with antral follicle counts (8), and establish its evidence-based diagnostic value for early intervention in primary care, two key gaps remain unresolved: the development of compatible reference standards to improve result comparability and the establishment of standardized ethnic-specific algorithms required for equitable clinical implementation (9).

Recent multiethnic studies reveal striking variations: Samoan women exhibit paradoxically high AMH levels (95th percentile: 7.02 ng/ml) independent of BMI (10), whereas East Asian women require 15%–20% lower thresholds (3.8–4.2 ng/ml) than Europeans (4.7–5.1 ng/ml) to avoid overdiagnosis, These differences mirror PCOS prevalence heterogeneity, ranging from 3.4% in Samoans to 7.3% in Chinese populations (11). while Middle Eastern women demonstrate accelerated AMH decline post-35 years (0.31 ng/ml/year) linked to vitamin D deficiency and consanguinity (12).

In addition to its diagnostic role, AMH is increasingly implicated in PCOS pathogenesis. It exacerbates follicular arrest by inhibiting follicular sensitivity to FSH and synergistically promotes anovulation with hyperandrogenism (13, 14). Beyond diagnostics, AMH actively perpetuates PCOS pathogenesis. It exacerbates follicular arrest via TGF-β/Smad-mediated aromatase suppression in granulosa cells, reducing estrogen synthesis (15), and synergizes with hyperandrogenism through an “AMH-LH positive feedback loop” in hypothalamic GnRH neurons, amplifying neuroendocrine dysfunction (16). Research shows that long-term exposure to high concentrations of PM2.5 (>50 μg/m^3^) inhibits the activity of ovarian tyrosine hydroxylase through oxidative stress, activates the HPA axis, disrupts the follicular development cycle, and disturbs endocrine and ovarian functions. The damage of PM2.5 to ovarian reserve is dose-dependent. For every increase of one IQR in concentration, the AMH level decreases by 10%. Younger women are more sensitive to PM2.5, and the decline of AMH is more significant, suggesting that early exposure may cause irreversible reproductive damage in the long term (17, 18). Metabolomic studies reveal that elevated AMH levels in PCOS patients correlate with reduced noradrenergic metabolite DOPGAL, potentially disrupting sympathetic modulation of follicular responsiveness and associating with steroidogenic pathway dysregulation in ovarian dysfunction (19). These findings underscore AMH's dual role as both a biomarker and a therapeutic target in PCOS.

While these advances highlight AMH's dual significance, critical translational gaps persist that warrant deeper investigation. Specifically, despite AMH's dual role as a biomarker and therapeutic target, critical knowledge gaps remain. Current studies predominantly focus on Caucasian populations, underrepresenting low-AMH ethnic groups like Indians (30% lower vs. Europeans) and Arabs (40.6% with AMH < 1.3 ng/ml) (20). No systematic synthesis exists on how sociocultural factors (e.g., consanguinity in 80% Middle Eastern marriages) or environmental determinants (e.g., air pollution reducing Chinese AMH by 28% vs. Caucasians) modulate PCOS-AMH dynamics (21). This bibliometric analysis aims to map global research trends, identify underserved populations, and highlight precision medicine opportunities in PCOS management.

This bibliometric analysis employs quantitative methods using CiteSpace and VOSviewer to map global research trends in Web of Science and PubMed publications (2000–2024), prioritizing three translational gaps: (1) ethnic disparities in AMH-metabolome interactions, (2) epigenetic regulation of AMH signaling, and (3) clinical validation of precision AMH thresholds. By synthesizing multidisciplinary evidence from genomics, public health, and molecular endocrinology, we aim to accelerate data-driven development of equitable PCOS diagnostic frameworks and therapeutic innovation.

## 2 Methods

### 2.1 Data collection

**Figure 1:**
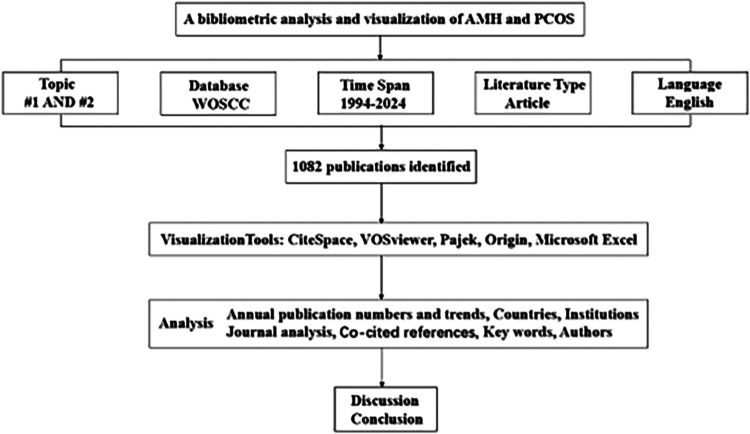
Flowchart of bibliometric analysis.

The Web of Science Core Collection (WOSCC) is an online database that provides high-quality, rapidly updated reference datasets for academic research (22). Among these databases, the Science Citation Index Expanded (SCI-E) (http://www.webofscience.com/) is recognised as the most widely used, authoritative, and highest standard database globally, making it one of the most suitable databases for bibliometric analysis (23). Adhering to the PRISMA guidelines, this study systematically searched for articles on AMH and PCOS research published in the SCI-E from 1994 to 2024. The publication type was limited to “article,” and the language was limited to English, yielding a final set of 1,082 articles. To ensure data accuracy and reliability, two authors independently conducted the literature search on October 1, 2024. Discrepancies were resolved through discussion with a third author or the entire research team. The search strategy included #1, which was AMH or its synonyms (TS); #2, which was PCOS or its synonyms (TS); and #3, which was “#1 AND #2” (the detailed search strategy is presented in Table 1). The flow diagram is depicted in Figure 1.

**Table 1:** Bibliometric analysis retrieval strategy for AMH and PCOS.

Strategy	#1	#2	#3
Words	TS = (Anti-Mullerian Hormone OR Anti Mullerian Hormone OR Antimullerian Hormone OR Mullerian Inhibiting Hormone OR Mullerian Inhibiting Substance OR Mullerian Regression Factor OR Mullerian-Inhibiting Hormone OR Mullerian-Inhibitory Substance OR Mullerian Inhibitory Substance OR Anti-Muellerian Hormone OR Anti Muellerian Hormone OR Anti-Mullerian Factor OR Anti Mullerian Factor OR Mullerian-Inhibiting Factor OR Mullerian Inhibiting Factor)	TS = (Polycystic Ovary Syndrome OR Polycystic Ovarian Syndrome OR Polycystic Ovary Syndrome 1 OR Sclerocystic Ovarian Degeneration OR Stein-Leventhal Syndrome OR Stein Leventhal Syndrome OR Sclerocystic Ovaries OR Sclerocystic Ovary)	#1 AND #2

### 2.2 Data analysis and visualization

Export the retrieved articles in plain text format, including complete records and references, and name the file “download_XX.txt”. Utilize Origin, Microsoft Excel, Pajek, as well as the visualization analysis software VOSviewer Version 1.6.20 and CiteSpace Version 6.2.R4, to conduct analyses on overall trends, countries and institutions, journals and cited literature, keyword co-citation bursts, and author publications. Present the analysis results graphically to explore the research hotspots, trends, and directions of AMH and PCOS over the past 30 years. While these conventional bibliometric methods provide valuable insights, they have limitations in trend prediction and semantic analysis. Future research could employ machine learning algorithms such as LSTM time series prediction and cluster analysis to uncover potential research trends or apply natural language processing (NLP) to analyze the semantic evolution of keywords. Additionally, integrating machine learning models to predict emerging research trends based on historical keyword co-occurrence patterns could enable proactive identification of translational gaps.

## 3 Results

### 3.1 Annual publication numbers and trends over the past 30 years

**Figure 2:**
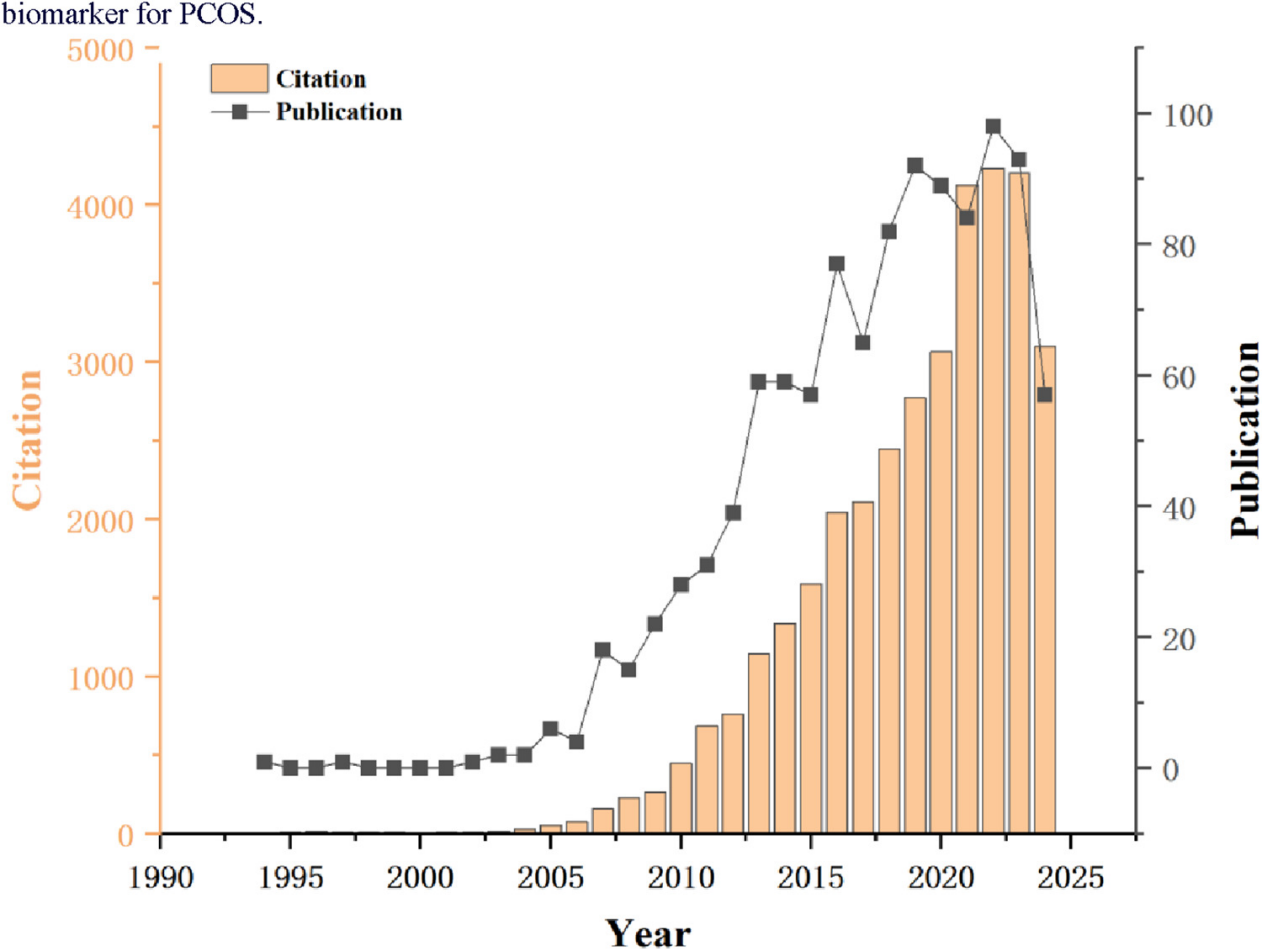
Annual trend of publications.

Based on the collected data, a total of 1,082 documents related to AMH and PCOS were retrieved from the WOSCC from 1994 to 2024. The distribution of annual publication volumes is illustrated in Figure 2. The figure reveals that between 1994 and 2006, the annual number of publications related to these studies was relatively low, followed by a steady growth trend. Notably, after 2012, the growth rate of publications accelerated, indicating a gradual increase in the emphasis on research into AMH as a biomarker for PCOS.

### 3.2 Countries

**Figure 3:**
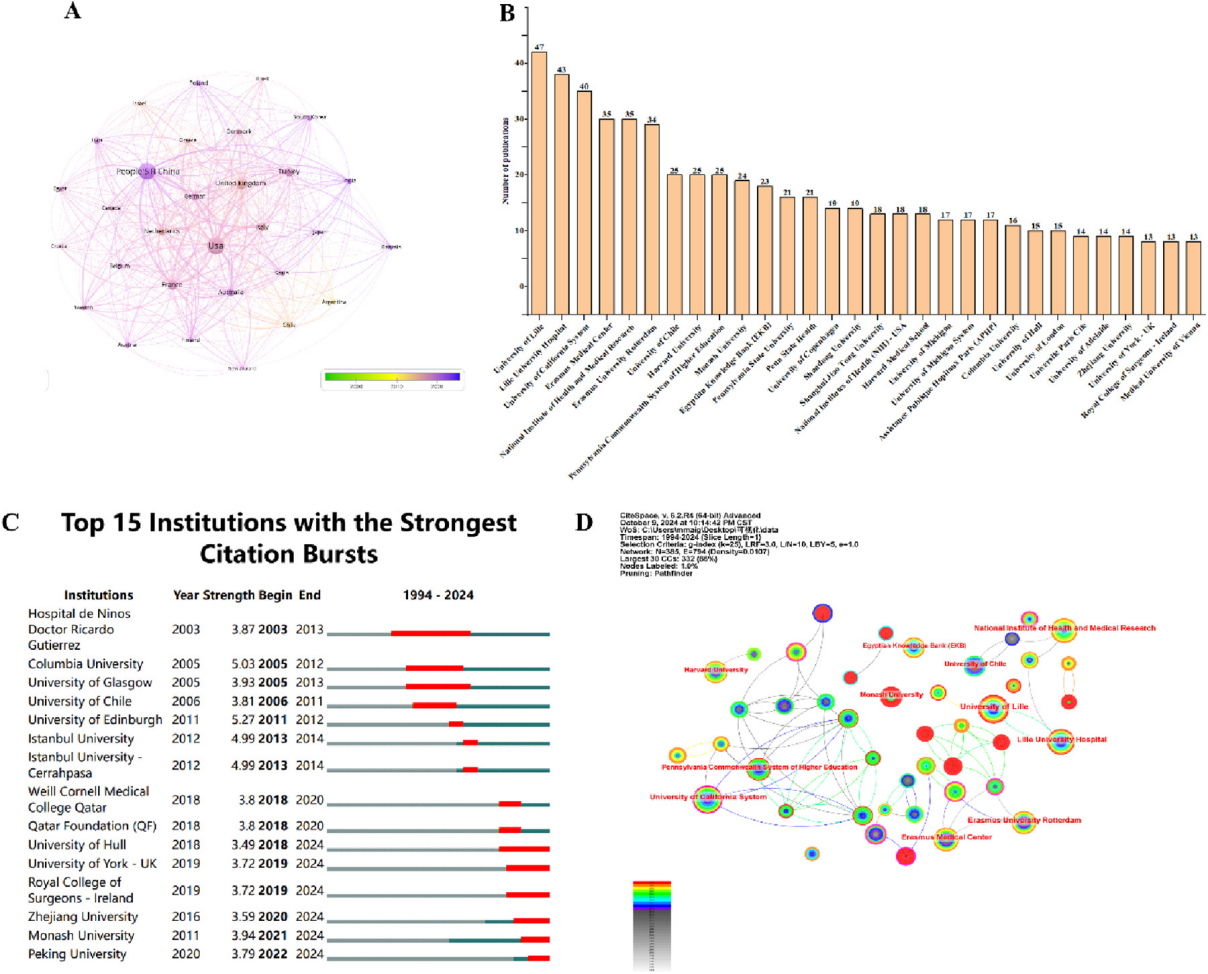
Publications of intuitions and countries. **(A)** A network visualization diagram based on the number of publications per country (*n* = 30, with one country having ≥10 publications). **(B)** The top 30 institutions by publication volume. **(C)** The top 15 institutions with citation bursts. **(D)** Institutions and their collaboration ties (the red-highlighted portion indicates institutions that have been active in recent years, *n* = 45, with one institution having ≥10 publications).

A total of 70 countries worldwide have made significant contributions to AMH and PCOS research. Table 2 lists the top 10 countries with the highest number of publications in this field. Among them, the United States has the largest number of publications, with 234 articles (21.6%) over the past 30 years. These publications have been cited a total of 12,742 times, averaging 54 citations per article. China ranks second with 227 articles (21%), receiving 3,911 citations and an average of 17 citations per article. The United Kingdom is ranked third with 106 articles (9.8%), followed by France with 83 articles (7.7%) and Turkey with 71 articles (6.6%). Upon examining the top 10 countries by publication volume, we found that although the Netherlands ranks lower, it has the highest average citation count (109). In contrast, China, which ranks second, has the lowest average citation count among the top 10 countries, suggesting that the research quality may be relatively lower than that of other countries. Figure 3A illustrates that 30 countries have published 10 or more articles in this field, and these countries exhibit research collaboration ties. The colour shade represents the publication timeline, whereas darker purple hues indicate more recent article publication dates.

**Table 2:** The top 10 countries in terms of publication volume.

Rank	Countries	Documents (*n*)	Percentage (*n*/1,082)	Citations
1	USA	234	21.6	12,742
2	People's R China	227	21	3,911
3	United Kingdom	106	9.8	4,650
4	France	83	7.7	6,787
5	Turkey	71	6.6	3,803
6	Australia	51	4.7	3,988
7	Italy	51	4.7	5,275
8	Netherlands	51	4.7	5,572
9	Germany	40	3.7	1,242
10	Iran	37	3.4	737

### 3.3 Institutions

Approximately 1,363 institutions globally have conducted bibliometric (LT) analyses on research related to AMH and PCOS. Figure 3B presents the top 30 institutions involved in this research field, along with their respective publication counts. Figure 3C shows that the institutions with the strongest citation bursts are Zhejiang University, Monash University, and Peking University. Table 3 provides detailed information on the top 10 institutions by publication output, including the number of publications and their respective percentages. Among the top 10 institutions, three are located in the United States and three in France, with the French institutions being more active. Université de Lille has the highest number of publications (*n* = 47, accounting for 3.4%), followed by Centre Hospitalier Universitaire de Lille (*n* = 43, accounting for 3.2%). The major institutions in this field and their collaboration ties are shown in Figure 3D. Co-occurrence analysis of these institutions reveals that those with higher publication volumes often have close collaboration ties, and the institutions with the most recent active publications are Monash University (*n* = 24), Columbia University (*n* = 16), University of Hull (*n* = 15), and Zhejiang University (*n* = 14).

**Table 3:** The top 10 institutions by publication volume.

Rank	Institutions	Countries	Publications	Percentage (*n*/1,363)
1	University of Lille	France	47	3.4
2	Lille University Hospital	France	43	3.2
3	University of California System	USA	40	2.9
4	Erasmus Medical Center	Netherlands	35	2.6
5	National Institute of Health and Medical	France	35	2.6
6	Research	Netherlands	34	2.5
7	Erasmus University Rotterdam	Chile	25	1.8
8	University of Chile	USA	25	1.8
9	Harvard University	USA	25	1.8
10	Pennsylvania Commonwealth System of Higher Education Monash University	Australia	24	1.8

### 3.4 Journal analysis

**Figure 4:**
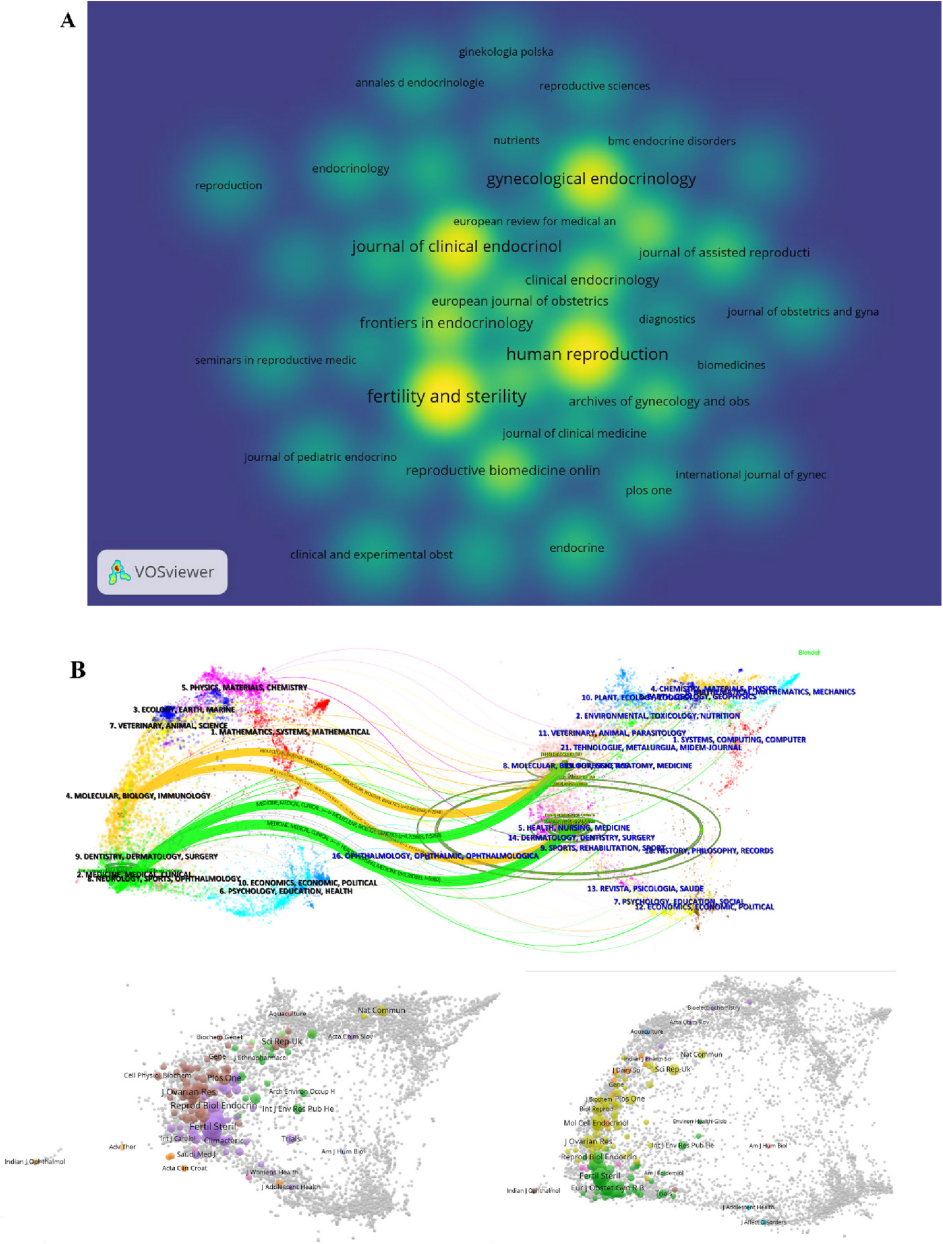
Journal analysis. **(A)** Top 37 journals by publication volume (*n* = 37, with each journal having ≥5 publications). **(B)** Journal overlay analysis.

A total of 264 journals worldwide are involved. Figure 4A displays the top 37 journals with the highest number of publications. Among them, *Fertility and Sterility* from the United States has published 98 articles with 5,802 citations and an impact factor of 6.7; *Human Reproduction* from the United Kingdom has published 88 articles with 4,286 citations and an impact factor of 6.1; *Journal of Clinical Endocrinology & Metabolism* from the United States has published 71 articles with 6,254 citations and an impact factor of 5.8; *Gynecological Endocrinology* from the United Kingdom has published 63 articles with 974 citations and an impact factor of 2.0; and *Frontiers in Endocrinology* from Switzerland has published 39 articles with 312 citations and an impact factor of 5.2. These figures indicate that articles published in these journals have significant academic influence. Figure 4B presents an overlay analysis of journals, examining the relationship between disciplinary categories in AMH and PCOS research. With the citing journals on the left of the map and the cited journals on the right, it depicts the distribution of the most frequently cited journals. The two green bars signify journals frequently cited in medical and clinical research, which may belong to fields such as health, nursing, genetics and molecular biology, biology, medicine, psychology, sociology, and education. The two yellow bars, in contrast, represent areas with less dense citation relationships and frequencies compared to those indicated by the green bars and also indicate emerging disciplines such as economics, politics, rehabilitation medicine, and philosophy. It is evident that both yellow and green bars originate from the two disciplinary fields on the right side, suggesting that the publication knowledge base related to AMH and PCOS is primarily based on the two disciplines on the right side of the map (“Healthcare medicine” and “molecular biology and genetics”).

### 3.5 Co-cited references

**Figure 5:**
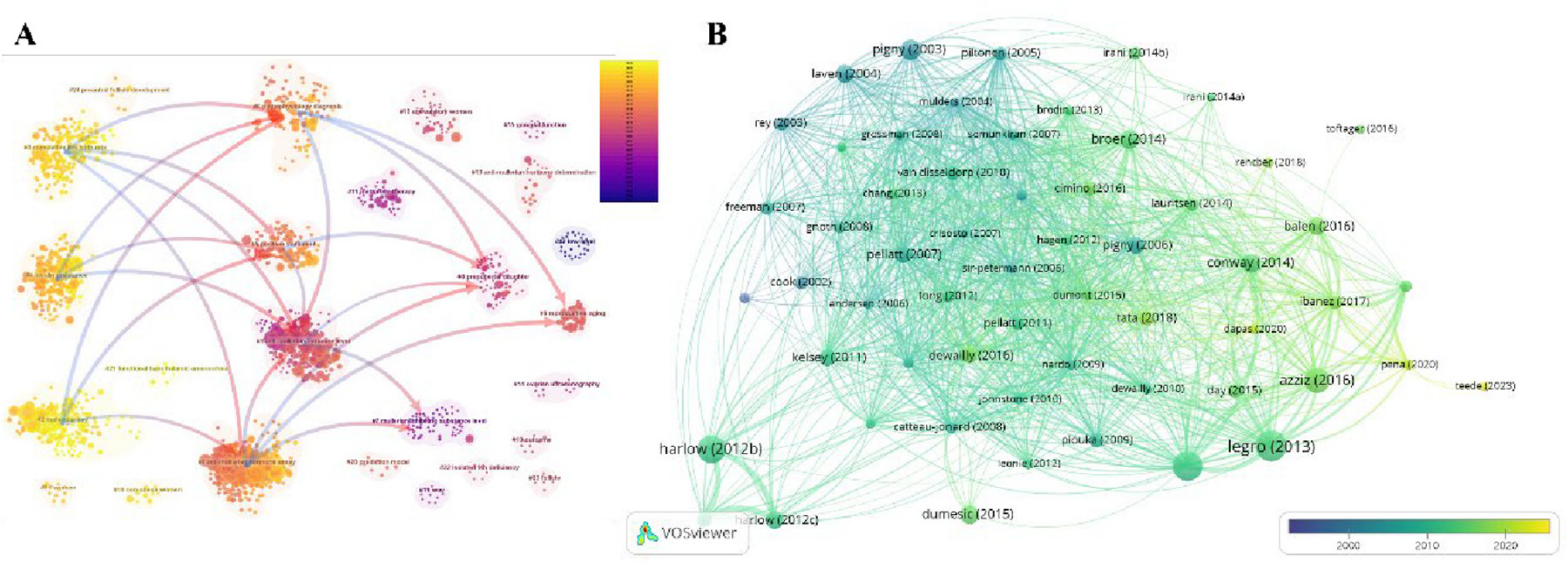
Co-cited references. **(A)** Cluster analysis of cited literature. **(B)** Collaboration network of cited literature (*n* = 58, with each individual literature cited ≥100 times).

As shown in Figure 5A, the cluster analysis of co-citation of references, the clusters labelled as “#2 molecular key,” “#3 cumulative live birth rate,” and “#4 insulin resistance” are newer and important co-citation analysis clusters. This indicates that research on AMH and PCOS focuses on these three areas: “molecular key,” “cumulative live birth rate,” and “insulin resistance,” spanning multiple fields such as molecular biology, reproductive health, and metabolic abnormalities. Additionally, Modularity *Q* is an indicator used to measure the quality of clustering, with values ranging from −0.5 to 1. A higher value indicates a more reasonable clustering result. The modularity *Q* value in this study is 0.749, indicating a high degree of internal consistency and separation in the clustering results. Weighted Mean Silhouette S is another indicator for evaluating the clustering effect, with values ranging from −1 to 1. A higher value indicates a higher degree of similarity within clusters and a higher degree of dissimilarity between clusters. The weighted average silhouette coefficient in this study is 0.8068, indicating an excellent clustering effect. Furthermore, Figure 5B displays the references cited more than 100 times, with the bolded Legro (2013) being the most frequently cited article.

### 3.6 Keywords

**Figure 6:**
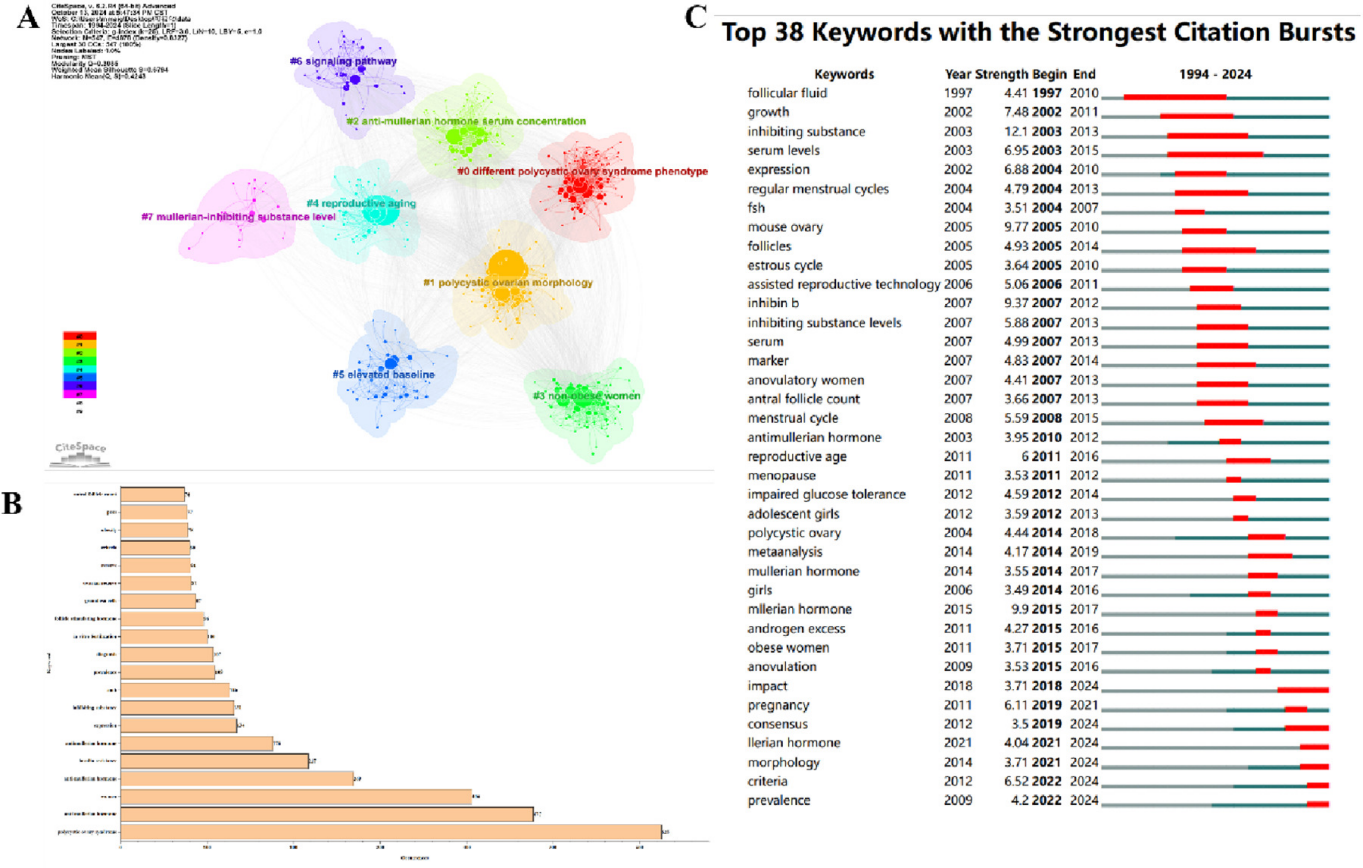
Keywords. **(A)** Keyword cluster analysis. **(B)** Top 20 keywords. **(C)** Keyword burst.

Figure 6A displays 547 keywords categorized into 8 clusters. The first cluster, #0 Different Polycystic Ovary Syndrome Phenotype, shown in red, focuses on the various phenotypes of PCOS. The second cluster, #1 Polycystic Ovarian Morphology, in yellow, emphasizes research on polycystic ovarian morphology. The third cluster, #2 Anti-Müllerian Hormone Serum Concentration, in light green, focuses on the serum concentration of AMH. The fourth cluster, #3 Non-Obese Women, in green, emphasizes research related to non-obese women. The fifth cluster, #4 Reproductive Aging, in light blue, focuses on the mechanisms of reproductive aging and how interventions can delay the aging process. The sixth cluster, #5 Elevated Baseline, in dark blue, focuses on the impact of elevated baseline levels on female reproductive health. The seventh cluster, #6 Signaling Pathway, in purple, focuses on research on signalling pathways. The eighth cluster, #7 Müllerian-Inhibiting Substance Level, in pink, emphasizes the level of Müllerian-inhibiting substance (MIS), studying its function in the female reproductive system and potential therapeutic applications. Early emerging keywords are shown in blue, while recent keywords are in red. Figure 6B lists the top 20 keywords sorted by frequency, with “polycystic ovary syndrome” being the most frequently used keyword, appearing 625 times, followed by “anti-Müllerian hormone” (*n* = 477) and “women” (*n* = 406). Figure 6C shows that in this research on AMH and PCOS, “consensus,” “morphology,” “criteria,” “prevalence,” and “Müllerian hormone” are keywords that continue to experience a surge in usage up to 2024.

### 3.7 Authors

**Figure 7:**
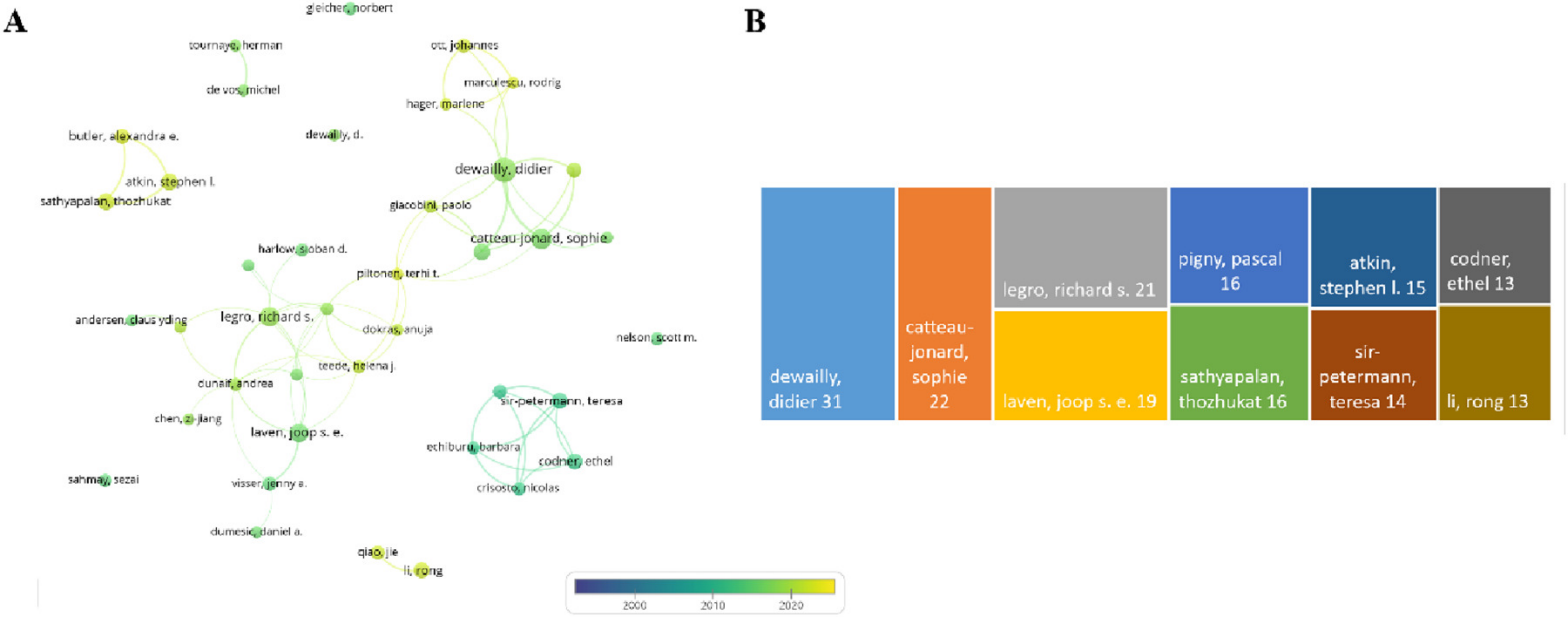
Author analysis. **(A)** Author collaboration network diagram. **(B)** Top 10 authors by publication volume.

Through the analysis of publications, a collaboration network was constructed based on authors with at least 8 publications. As illustrated in Figure 7A, this network encompasses English publications from 40 authors, among which the nodes representing “dewailly, didier,” “catteau-jonard, sophie,” and “legro, richard s.” are the largest, indicating that they have authored the highest number of papers. Figure 7B reveals that Dewailly, Didier, affiliated with the Centre Hospitalier Régional Universitaire de Lille, Division of Reproductive Endocrinology, has been dedicated to research in this field and has published 31 articles.

## 4 Discussion

Stein and Leventhal were the first to describe PCOS as a syndrome of bilateral polycystic ovarian enlargement associated with anovulation, potentially accompanied by metabolic disturbances and psychological disorders, which was later named Stein-Leventhal syndrome (S-L syndrome) (24–27). In 1990, the National Institutes of Health (NIH) established diagnostic criteria for PCOS, which included menstrual irregularities and anovulation, clinical or biochemical evidence of hyperandrogenism, and the exclusion of other causes of hyperandrogenism. At that time, polycystic ovarian changes (PCO) were not considered a primary diagnostic criterion (28). In 2003, the European Society of Human Reproduction and Embryology (ESHRE) and the American Society for Reproductive Medicine (ASRM) convened an expert meeting in Rotterdam, Netherlands, to establish international diagnostic criteria for PCOS, known as the Rotterdam criteria. These criteria include oligo- or anovulation, clinical and/or biochemical signs of hyperandrogenism, and polycystic ovaries on ultrasound. A diagnosis of PCOS can be made if two of these criteria are met and other causes are excluded (29). This Rotterdam criterion has been widely adopted in the subsequent 20 years. During this period, although the Androgen Excess Society (AES), the Chinese Ministry of Health, and the International Evidence-Based Guidelines, among others, have proposed their respective recommendations for PCOS diagnostic criteria, their popularity and widespread acceptance—except for the Rotterdam criteria—have been limited (30–32). In 2023, ESHRE, the American Society for Reproductive Medicine (ASRM), the Endocrine Society of the United States (USA), and the European Society of Endocrinology published an updated version of the International Evidence-Based Guidelines for PCOS, These guidelines did not overturn the Rotterdam criteria but, for the first time, explicitly incorporated serum AMH testing into the diagnostic system. When ultrasound assessment is limited. AMH levels can be used as a surrogate indicator of polycystic ovarian morphology (33). The annual publication trend shows a steady increase in the number of relevant studies, which may be closely related to the continuous updating of the diagnostic criteria for PCOS and the deepening of the understanding of the biological value of AMH. This change marks a critical leap forward for AMH from basic research to clinical practice. AMH detection has the characteristics of high standardization and small periodic fluctuation, and it is especially suitable for obese patients with ovarian imaging difficulties or immature reproductive organs of adolescents. In terms of the evidence base, the literature metrics for this study show a surge in studies related to AMH and PCOS diagnosis after 2015, which is consistent with multicenter clinical validation studies.(e.g., AMH ≥4.7 ng/ml has a diagnostic sensitivity of 92% for PCOS (34). However, challenges remain: although AMH is recognized by guidelines, its full clinical application is still hindered: threshold standardization is missing, and there are population differences in AMH diagnostic thresholds at present (e.g., the threshold for East Asian women is about 12%–20% lower than that in Europe (35), but global uniform standards have not been established; Additionally, heterogeneity in testing techniques and bias between systems from different vendors may affect the comparability of results; there is also ongoing debate regarding cost-effectiveness, AMH testing in resource-limited areas (approx.$40–80/session) compared to ultrasound ($10-$30/session) remains to be validated. Future directions include optimizing diagnostic systems using race-stratified AMH thresholds, for example, East Asian vs. European women, and dynamic monitoring to guide ovulation induction therapy.

AMH's clinical use faces technical and contextual challenges. Its role in adolescent PCOS diagnosis is controversial due to physiological fluctuations. Combining AMH with metabolic markers (e.g., HOMA-IR) may improve specificity (36). A suggested algorithm for Chinese adolescents is AMH ≥6.32 ng/ml to reduce misdiagnosis (37). In obese patients with difficult ovarian imaging, AMH serves as a reliable PCOM surrogate. In resource-limited areas, AMH testing is a cost-effective first-line tool, followed by selective ultrasound for borderline cases. AMH also predicts ovulation induction outcomes (38). In PCOS women, AMH ≤16.43 ng/ml predicts letrozole-induced ovulation success, higher than clomiphene, showing AMH specificity in drug response (39). Elecsys® assays produce lower AMH values than Gen II, but they demonstrate comparable diagnostic performance for PCOM with method-specific thresholds, Therefore, standardization of AMH measurements is crucial (40).

Despite the advancements in diagnostic criteria and the growing recognition of AMH as a valuable biomarker, the selection of biomarkers for PCOS diagnosis remains a critical challenge (41). Among the commonly used biomarkers, AMH demonstrates superior stability and predictive value in quantifying ovarian reserve independently of the menstrual cycle, in contrast to SHBG, which is influenced by metabolic factors, and androstenedione, which exhibits cyclic variability (42–44). However, the clinical application of AMH is limited by standardization issues. For instance, the systematic overestimation of AMH levels by the Gen II assay underscores methodological differences across different platforms (40, 45). Additionally, there is a lack of established thresholds for specific populations (46, 47). Studies have shown that South Asian women have a higher serum AMH level compared with white European and Afro-Caribbean women (*F* = 3.817; *P* < 0.005), indicating a potential need for further exploration of whether race-stratified diagnostic criteria are necessary (35).

In terms of the primary countries contributing to research on AMH and PCOS, the United States leads with 234 publications, demonstrating its strong research resources and team capabilities. China follows closely with 227 publications, reflecting the country's emphasis on this field and significant growth in research output in recent years. However, there is a notable difference in average citation counts between China and the United States, suggesting that China still needs to improve in terms of research depth and impact. Although the Netherlands ranks lower in publication volume, it has the highest average citation count, indicating the high quality and international recognition of its research. Furthermore, an in-depth analysis of collaboration among institutions reveals that top-ranked institutions collaborate with each other. However, when this collaboration extends to the international level, these institutions rarely participate in cooperative networks with institutions from other countries, preferring instead to collaborate domestically. This observation indicates that the current global collaboration landscape for research on AMH and PCOS is relatively narrow, with a lack of breadth in research collaboration networks. There is some geographical concentration in research collaboration, with a more prominent limitation of collaboration across continents. As international collaboration has become a common phenomenon in many scientific research fields, countries should strengthen international cooperation, share resources, exchange experiences, and jointly advance research development.

The most frequently cited article is “Diagnosis and treatment of polycystic ovary syndrome: an Endocrine Society clinical practice guideline.” It's a comprehensive and systematic analysis of the diagnosis, complication assessment, and treatment of PCOS, which carries significant authority and consensus. It is supported by the consensus statement of the Androgen Excess and Polycystic Ovary Syndrome Society. Although the article was published some time ago, research in the field of PCOS continues to progress. As a seminal work in this field, the citation count of this article is likely to continue to increase with the emergence of new studies.

Focusing on key researchers who have made significant academic contributions in this field, we identify three authors in particular: Dewailly, Didier, Catteau-Jonard, Sophie, and Legro, Richard S. Their research outputs are prominent in quantity. Among them, Dewailly, Didier's sustained research efforts and high publication volume in this field not only underscore his professional leadership but also indicate the importance of his institution in PCOS research. The co-authorship network shows that cross-country collaboration fosters academic exchange and cooperation globally. Additionally, the academic achievements of these authors reflect the research strength and direction in their respective regions. Therefore, research policies, funding allocation, academic orientations, and research quality are all crucial.

Finally, there has been significant growth in citations for keywords such as “consensus,” “morphology,” “criteria,” “prevalence,” and “Müllerian hormone.” This trend indicates that future research in the field of AMH and PCOS will place greater emphasis on strengthening consensus, deepening morphological studies, refining diagnostic criteria, monitoring prevalence trends, and exploring the therapeutic potential of AMH. At the same time, morphological research will further investigate the intrinsic relationship between ovarian morphological changes and AMH levels to further understand the role of AMH in PCOS. Additionally, as research progresses, diagnostic criteria for PCOS will continue to be refined, and AMH levels, as an important indicator formulation, will be more widely used in diagnosis to enhance diagnostic accuracy. Researchers will also continue to monitor trends in the prevalence of PCOS to provide a scientific basis for formulating effective prevention and control strategies. Lastly, the complex mechanisms of action of AMH in PCOS hold the potential for developing new therapeutic approaches and driving advancements in research and clinical applications in this field.

The present study has the following limitations. Firstly, reliance on the WoS CC database limits coverage of non-English and regional journals, such as China's CNKI and Latin America's SciELO, potentially omitting crucial regional findings, like AMH threshold studies in East Asian populations. Future research should integrate multiple databases, including Scopus and PubMed, to enhance global representativeness and reduce misjudgment of AMH's efficacy as a PCOS biomarker due to data bias. Secondly, the lag in literature inclusion hinders real-time tracking of AMH-related innovations, such as advancements in detection technology and updates to diagnostic criteria, as well as the capture of emerging interdisciplinary trends, such as the interaction between AMH and adolescent development. This results in conservative hotspot assessments and difficulty in dynamically presenting cutting-edge progress in the field. Thirdly, the association between AMH and PCOS exhibits significant racial and regional differences. Failure to adequately distinguish population biological characteristics in studies may lead to the generalization of AMH diagnostic thresholds across regions. Meanwhile, controversies in adolescent PCOS diagnosis, such as confounding effects with precocious puberty, relying excessively on European and American cohorts while neglecting validation in non-Western adolescent populations, will compromise the universality of conclusions. Fourthly, AMH's diagnostic complexity persists, particularly in adolescents and older patients. In obese adolescents without PCOS, AMH levels may also increase, further reducing its diagnostic specificity. Physiological fluctuations in AMH levels overlap with PCOS phenotypes, raising overdiagnosis risks (48, 49). In pregnant women with PCOS, AMH levels remain elevated, showing greater sensitivity and specificity compared to other biomarkers. Combined with age-related ovarian decline, this complicates AMH interpretation in clinical settings (50, 51).

Currently, the diagnostic criteria for PCOS remain controversial, particularly in adolescents and perimenopausal women. Although biomarkers such as AMH show potential in PCOS diagnosis, their clinical application requires further validation. To achieve standardization of AMH testing, the following systematic steps must be implemented: Firstly, evaluate the bioactivity, stability, and immunoreactivity of recombinant human AMH across different detection methods. Secondly, establish a consensus value for AMH in international units (IU) through multi-laboratory collaborative studies, ensuring traceability of its mass units to SI units via physicochemical reference methods under the ISO 17511 guidelines. Additionally, explores the feasibility of using natural human AMH serum as an international standard while addressing challenges such as heterogeneity. This will significantly change practice and offer women a low-cost, convenient option, without evidence of overdiagnosis (52). Cost-benefit analyses further support this transition: AMH testing (∼$40–80/test) demonstrates 28% higher cost-effectiveness compared to routine ultrasound (∼$10–30/session) in low-resource settings when considering reduced misdiagnosis rates and streamlined workflows. Furthermore, the standardization framework must be continuously refined by integrating novel detection technologies through multicenter collaboration and data sharing, thereby ensuring accuracy and clinical reliability (53).

In clinical practice, PCOS diagnosis should involve a multidimensional assessment, including hormone levels such as androstenedione and SHBG, ovarian ultrasonographic morphology, metabolic indicators such as insulin resistance and lipid profiles, clinical manifestations such as hyperandrogenism and menstrual irregularities, and medical history (54). To optimize resource allocation, we propose a two-stage diagnostic algorithm: initial screening using standardized AMH thresholds, with confirmatory ultrasound or metabolic profiling for borderline cases. Additionally, as a supplementary biomarker, AMH requires standardized age—and ethnicity-stratified thresholds to minimize misdiagnosis risks. This phased approach not only reduces unnecessary imaging in adolescents and obese populations but also aligns with precision medicine goals by prioritizing biomarker-guided stratification. Future research should focus on elucidating the pathological mechanisms of PCOS, validating the synergistic diagnostic value of AMH with other biomarkers such as miRNAs and metabolomic markers, and enhancing diagnostic precision through advanced technologies. Current emerging artificial intelligence has a key role in this process; it is expected to develop multicenter AMH databases combined with AI algorithms to dynamically calibrate race-specific thresholds. In addition, generative AI can accelerate the integration of interdisciplinary knowledge and ultimately advance the development of personalized diagnostic and therapeutic frameworks.

## 5 Conclusion

This study provides a comprehensive analysis of research in the field of AMH and PCOS using bibliometric methods, revealing the current research status, hotspots, and trends in this field. At present, there is no specific diagnostic test for PCOS, and its diagnosis primarily relies on ultrasound examination showing hyperandrogenism, menstrual abnormalities, and PCOM. Therefore, there is an urgent need to develop an efficient, standardized, and cost-effective diagnostic method for identifying PCOS. The research results indicate that AMH, as a biomarker for PCOS, has garnered widespread attention in research on diagnosis, treatment, and pathogenesis. Future studies are expected to further explore the clinical application of AMH and its combined use with other biomarkers, providing more scientific evidence for the precision treatment of PCOS.

## Data Availability

Publicly available datasets were analyzed in this study. This data can be found here: Science Citation Index Expanded (SCI-E) (http://www.webofscience.com/).
